# Antibiofilm action of ozonated sunflower oil against bacteria isolated from the uterus of mares susceptible to endometritis

**DOI:** 10.1590/1984-3143-AR2025-0116

**Published:** 2026-07-06

**Authors:** Antonio Brito da Silva, Laís Querino Barbosa Freire, Maria Eduarda Pedrosa Leite, Lamartine Rodrigues Martins, Gilvannya Gonçalves de Sobral, Marcelo Mendonça, Elizabete Rodrigues da Silva, Haroldo Vargas Leal, Gustavo Ferrer Carneiro

**Affiliations:** 1 Departamento de Medicina Veterinária, Universidade Federal Rural de Pernambuco – UFRPE, Recife, PE, Brasil; 2 Laboratório de Reprodução Animal de Pernambuco, Universidade Federal do Agreste de Pernambuco – UFAPE, Garanhuns, PE, Brasil; 3 Universidade Federal de Pernambuco – UFPE, Recife, PE, Brasil; 4 Laboratório de Microbiologia e Imunologia, Universidade Federal do Agreste de Pernambuco – UFAPE, Garanhuns, PE, Brasil; 5 Laboratório de Microbiologia, Universidade Federal do Agreste de Pernambuco – UFAPE, Garanhuns, PE, Brasil; 6 Horses Vet. Services, Juiz de Fora, MG, Brasil

**Keywords:** ozone, endometritis, peroxide, reproduction, equines

## Abstract

This study aimed to characterize the antibiofilm action of ozone-enriched sunflower oil (O_3_) against pathogens associated with endometritis in mares. Initially, samples from 41 mares with a history of subfertility were stored in BHI broth and 0.9% NaCl solution. The samples underwent microbial culture, identification, and *in vitro* sensitivity testing at LABRAPE-UFRPE, Brazil. Biofilm production was evaluated by the crystal violet method. Subsequently, biofilm-forming bacteria were subjected to the action of different concentrations of ozonated sunflower oil. Of the 21 isolates tested for biofilm production, 76.2% (16/21) showed positive production, with 43.75% (7/16) being weak producers, 31.25% (5/16) moderate producers, and 25% (4/16) strong producers. After the action of ozonated sunflower oil at a concentration of 203.43 mEq/L, a reduction was observed in the mass of the gram-negative bacilli biofilm, however, no statistical difference was found for the gram-positive cocci biofilm. The results show that ozonated sunflower oil presented satisfactory results in breaking down gram-negative bacteria biofilm.

## Introduction

Among the illnesses that affect the reproductive system of equine females, endometritis is considered one of the most important (Troedsson, 2011; Causey et al., 2006; Leblanc et al., 2007; Overbeck et al., 2011; Woodward et al., 2013; Kozdrowski et al., 2015). Alterations caused to the uterine endometrium from this infectious or inflammatory process can lead to an inability to conceive, early embryonic death, and shortening of the luteal phase (Thomassian, 2007). Affected animals exhibit an irregular estrous cycle, require intensive management, and need a higher number of estrous cycles to achieve a viable product, which results in increased production costs (Diel de Amorim et al., 2015).

A positive correlation has been reported between chronic uterine infections in mares that do not respond to antibiotic treatment and bacterial isolates capable of producing biofilms *in vitro* (Vargas et al., 2019). A biofilm consists of a matrix of polysaccharides synthesized by microbial communities, adhered to biotic or abiotic surfaces, which hinders the action of antibiotics (Donlan and Costerton, 2002; Patra et al., 2025). Experimental evidence demonstrates that gram-negative bacteria isolated from the uterus of mares are capable of forming biofilms *in vitro*, and that *Pseudomonas aeruginosa* can form biofilms in the endometrium, locally modulating the host's immune response. Furthermore, bacteria such as *Escherichia coli* can remain associated with the epithelial surface under biofilm coverage, which can hinder their detection and contribute to the persistence of infection (Ferris et al., 2016, 2017; Tukia et al., 2023).

The therapeutic use of ozone (O_3_) began more than 150 years ago (Elvis and Ekta, 2011). It was first used as a microbicidal molecule in 1856 and again in 1860 to sterilize surgical rooms and water treatment systems, respectively (Merhi et al., 2019). Recent studies demonstrate that O_3_ possesses antioxidant, anti-inflammatory, immunostimulatory, and angiogenic effects, with minimal side effects, particularly in equine reproduction (Ferreira et al., 2021; Almeida et al., 2021).

The ozonolysis of sunflower oil proceeds via the classical Criegee mechanism, acting on the double bonds of unsaturated fatty acyl chains, mainly oleic and linoleic residues (Criegee, 1975). Initially, O_3_ reacts with the carbon–carbon double bond to form an unstable primary ozonide, which subsequently recombines with carbonyl species to generate more stable secondary ozonides, peroxides, and low‑molecular‑weight cleavage products, that progressively increase the oxidative and peroxide indices of the sunflower oil matrix (Bailey, 1978; Ledea, 2003).

Ozonated vegetable oils represent a more stable and practical form of ozone delivery compared with gaseous O_3_. During the ozonolysis of unsaturated fatty acids, ozone reacts with double bonds, forming relatively stable secondary ozonides, hydroperoxides, and other oxygenated compounds, that act as reservoirs capable of gradually releasing reactive oxygen species. These products confer antimicrobial and therapeutic properties to ozonated oils and have demonstrated activity against a wide range of bacterial and fungal pathogens (Repciuc et al., 2025; Vieira et al., 2025).

The action of ozonized oils in the degradation of biofilm biomass in microbial isolates has been evidenced in different contexts. Gentili et al. (2023) observed a significant reduction in biofilm formation and bacterial adhesion in human corneal cells. Similarly, Silva et al. (2020) verified high efficiency in the removal of methicillin-resistant *Staphylococcus aureus* biofilms in foot ulcers of diabetic patients. Furthermore, ozonized oil has been shown to exhibit effective antimicrobial activity against Candida species and *Streptococcus mutans* biofilm-derived cells in a murine model, with minimal toxicity (Higa et al., 2022). However, to date, few studies have investigated the efficacy of ozonized oils in the treatment of uterine biofilms in mares. Therefore, the present study aimed to evaluate the efficiency of ozonized sunflower oil in the degradation of biofilm produced in vitro by bacteria isolated from the uterus of susceptible mares.

## Methods

### Animals

This study included 41 mares, aged 4-26 years (11.0 ± 0.9), of different breeds, that had experienced failures during embryo collection or not become pregnant for three consecutive estrous cycles, originating from farms in the Agreste region of the state of Pernambuco, during the 2019 breeding season. The use of the animals in the study was previously authorized by the Ethics Committee on Animal Use of UFRPE, according to protocol 23082.008142/2019-76.

The animals were clinically evaluated to confirm signs of persistent endometritis. The gynecological evaluation consisted of observing the vulvar conformation, closure of the vulvar lips, presence of vulvar discharge, and palpation, followed by transrectal ultrasonography to determine the size, symmetry, presence or absence of content, consistency, and tubularity of the uterus, as well as the characteristics of the ovaries. Uterine samples were also collected for cytology and culture, and mares showing 2% polymorphonuclear cells in cytology and/or bacterial growth in culture were considered positive for persistent endometritis.

### Sample collection

After emptying the rectum with the aid of a transrectal palpation glove, the perianal and perivaginal regions were thoroughly cleaned with 2% chlorhexidine. The double-lumen cytology brush (PROVAR®) was then introduced, protected by the hand. The brush was inserted through the cervix with the aid of a finger, followed by the rupture of the first plastic protection and exposure of the collection set, already inserted into the uterus transcervically. The outer seal was then broken to allow the cytology brush to come into contact with the inner wall of the uterus, with gentle rotating movements. After sample collection, the inner rod was retracted into the outer tube and, after complete removal of the set from the uterus, the sample was transferred to a sterile bench. With the aid of a Bunsen burner, a sterile cytology slide was seeded, and then a sterile swab was rubbed on the cytology brush to obtain an aliquot of the sample for microbiological culture. These samples were stored in sterile glass test tubes containing brain-heart infusion broth (BHI). Samples from each mare were properly identified, stored in an expanded polystyrene box under refrigeration, and sent to the Animal Reproduction Laboratory of Pernambuco (LABRAPE) at UFRPE-UAG for culture analysis, bacterial identification, biofilm formation assessment, and determination of the minimum inhibitory concentration (MIC) and minimum bactericidal concentration (MBC) of ozonized sunflower oil, as well as its action on the bacterial biofilm.

### Bacterial identification

Samples in BHI broth were incubated in an aerobic oven at 36±1 °C for 24 h and subsequently seeded onto plates containing 6% (v/v) sheep blood agar and MacConkey agar and incubated again in an aerobic oven at 36±1 °C for up to 48 h. After the incubation period, the growth characteristics of the colonies on the plates were evaluated, including hemolysis production, pigmentation, morphology, and staining characteristics. Slides were then prepared using the Gram staining method, according to Quinn et al. (1994), to differentiate between gram-positive and gram-negative cocci and bacilli. All slides were subsequently subjected to the catalase assay.

The following biochemical assays were used to identify enterobacteria (catalase-negative gram-negative bacilli): urease production, nitrate broth, MR/VP (MR - reaction with methyl red; VP - Voges-Proskauer reaction), the SIM agar test (S - H_2_S production; I - indole production; M - motility), and citrate agar test (using carbon citrate). The bacteria were then identified according to the methodology employed by Carter (1988) and Quinn et al. (1994). The following tests were used to identify catalase-positive gram-positive cocci: the bacitracin and novobiocin test; coagulase test; and CAMP (Christie-Atkins-Munch-Peterson) test, according to Quinn et al. (1994).

### Antimicrobial susceptibility assay of planktonic bacteria

The MIC and MBC of the ozonated sunflower oil were determined through the microdilution method, as described in the guidelines of the Clinical and Laboratory Standards Institute (CLSI, 2024), with some modifications, using Mueller-Hinton broth with 0.5% Tween 80. Tween 80 was used to facilitate dilution of the ozonated sunflower oil into Mueller-Hinton broth as it is a liquid medium. To this end, ozonated sunflower oil with 406.87 mEq peroxide/kg index was used. A 1 mL aliquot of the suspension of each bacterial isolate adjusted to DO_600_ = 0.5 was distributed into five sterile glass micro-coagulation tubes. Next, 1 mL of ozonated sunflower oil was added to each tube for each bacterial isolate, at concentrations between 12.71 and 203.43 mEq peroxide/kg, with homogenization of the oil and the medium at each dilution, using Tween 80 as an emulsifier. After inoculation, the samples were incubated at 36±1 °C for 24 h under stirring. The MIC is the lowest concentration capable of inhibiting bacterial growth, which was determined by observing the tubes in a luminous environment compared to the negative control, while the MBC is the lowest concentration capable of inhibiting 99.9% of the bacteria in 24 hours. To determine the MBC, each MIC tube was sown onto plates with plate count agar (PCA), followed by bacterial count after incubation at 36±1 °C for 24 h. Ozonated sunflower oil in Mueller-Hinton broth with 0.5% Tween 80 and ozonated sunflower oil at the same dilutions were used as negative controls. These assays were performed in triplicate.

### Biofilm biomass quantification

The crystal violet method was employed to quantify bacterial biofilm formation, following the recommendations described by Darwish and Asfour (2013) and Melo et al. (2013). Briefly, three colonies of each bacterial isolate were inoculated in 3 mL glycosylated Tryptic Soy Broth (TSB) and incubated at 36±1 °C for 24 h. Next, each inoculum was diluted to 1:40 in sterile glycosylated TSB and 200 mL of the dilution of each sample were used to fill 96-well polystyrene microplates (CELLSTAR® Cell Culture Microplates, Greiner Bio-one, Germany), with five replicates per sample. After incubation at 36±1 °C for 24 h, the medium was discarded, the plates were washed three times with 250 µL 0.8% (w/v) saline solution, and dried through inversion at 36±1 °C for 1 h. Next, 200 µL of methanol were poured into each well to fixate the biofilm, which was discarded by inversion after 15 min at room temperature. Subsequently, 160 µL of the 0.5% (w/v) crystal violet solution were poured into each well before being discarded by inversion after 1 min. The plates were rinsed in distilled water until full removal of the dye. After that, 160 µL 95% ethanol were added to each well and immediately discarded. The plates were then dried at room temperature and read in a spectrophotometer at 570 nm.

Results were interpreted according to the criteria suggested by Darwish and Asfour (2013). After establishment of the cut-off point based on the arithmetic mean and standard deviation of the optical densities (ODs) of the negative control (ODc), the arithmetic mean of the ODs of the test samples (ODt) was used to classify the sample according to the following categories: Non-biofilm producer = ODt ≤ 0.06; weak producer = 0.06 < ODt ≤ 0.120; moderate producer = 0.120 < ODt ≤ 0.240; and strong biofilm producer = ODt > 0.240.

### Antibiofilm action of the sunflower ozonized oil

The biofilm-producing bacteria were again induced to production, as described above. After 24 h of incubation, the liquid content of the plate was removed and 200 µL of ozonated sunflower oil solution containing glycosylated TSB with 0.5% Tween 80 were added to five wells for each sample, to achieve the concentration of 203.43 mEq peroxide/kg. The same procedure was carried out in another plate for the negative control and, after induction of biofilm production, 200 µL of extra-virgin sunflower oil were added. The plates were then incubated for 24 h at 36±1 °C under stirring, after which the wells were stained with 0.5% crystal violet, before being dried and read in a spectrophotometer at 570 nm.

### Statistical analysis

To evaluate the influence of ozonated sunflower oil on the disruption of biofilms in gram-negative bacteria, the data were normalized and subjected to the Shapiro-Wilk and Skewness/Kurtosis tests, with p-values of 0.76 and 0.73, respectively (p > 0.05), indicating normal distribution. Subsequently, analysis of variance (ANOVA) was performed, revealing significant differences between groups. Group means were compared using Tukey’s post hoc test (p < 0.05). For gram-positive bacteria, the group mean data did not meet the assumptions of normality and homoscedasticity; therefore, nonparametric analysis was conducted using the Kruskal-Wallis test followed by Dunn’s post hoc test with the Bonferroni-Holm correction.

## Results

Microbiological analysis revealed a predominance of *Staphylococcus* spp., isolated in 25.8% (8/31) of the positive samples, followed by non-lactose fermenting gram-negative bacilli, identified in 12.9% (4/31). Microorganisms such as *β-hemolytic Streptococcus*, *Escherichia coli*, *Klebsiella* spp., and *Proteus* spp. were detected in equal proportions, each corresponding to 3.22% (1/31) of the samples. In addition, growth of bacteria of the genus *Bacillus* spp. was observed in 22.6% (7/31) of the samples and *Micrococcus* spp. in 6.45% (2/31), both considered contaminants due to their environmental nature. In addition, 12.9% (4/31) of cases presented mixed infection, evidencing the occurrence of more than one etiological agent in the same infectious condition. These results indicate a higher frequency of bacterial isolations, with a predominance of gram-positive cocci, reinforcing the potential role of *Staphylococcus* spp. as an agent associated with uterine infections in mares.

Evaluation of the antimicrobial activity of ozonated sunflower oil demonstrated considerable efficacy against pathogens isolated from mares with endometritis (Table 1). For gram-positive cocci and most gram-negative bacilli, a minimum inhibitory concentration (MIC) of 101.71 mEq of peroxide/kg was observed, while the minimum bactericidal concentration (MBC) was 203.43 mEq of peroxide/kg, indicating a constant 2:1 ratio between MBC and MIC. This pattern suggests that the oil exhibits effective bactericidal action at concentrations relatively close to those required to inhibit microbial growth. However, for *Proteus* spp., a lower MIC was observed (50.85 mEq of peroxide/kg), and the MBC was four times higher.

**Table 1:** Susceptibility of bacteria isolated from the uteruses of mares to different concentrations of ozonated sunflower oil.

**Bacterial class or genus**	**MIC**	**MBC**	**MBC:MIC**
	**(mEq peroxide/kg)**	**(mEq peroxide/kg)**	
**Gram-positive cocci**			
*Staphylococcus* spp*.*	101.77	203.43	2
β*-*hemolytic *Streptococcus*			
**Gram-negative bacilli**			
*E. coli*	102.77	203.43	2
*Klebsiella* spp.			
NLF gram-negative bacilli			
*Proteus spp.*	50.85	203.43	4

MIC: minimum inhibitory concentration; MBC: minimum bactericidal concentration; NFL: non-lactose-fermenting.

**Figure 1:**
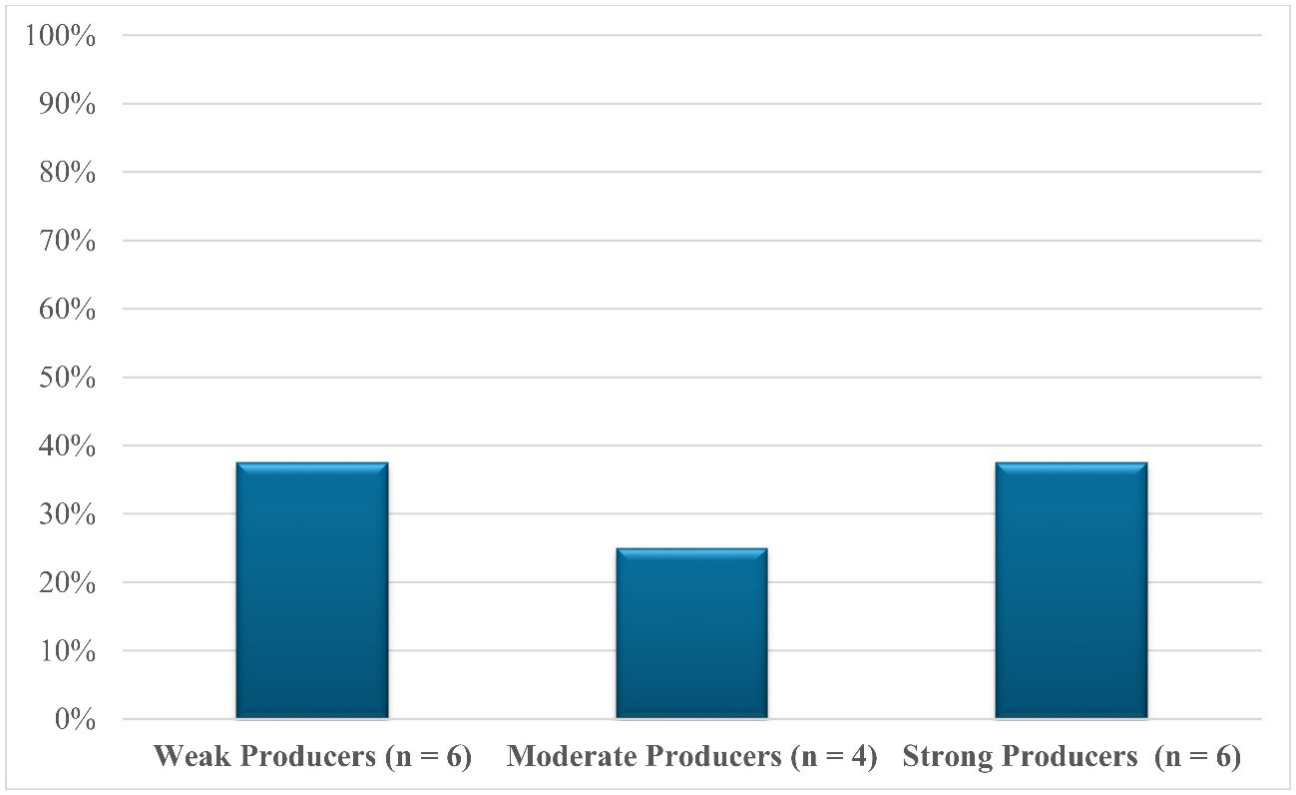
Classification of samples of bacteria isolated from the uteruses of susceptible mares, considering their *in vitro* biofilm production capacity.

Regarding biofilm production capacity, 76.2% (16/21) of the isolates tested showed a positive result, revealing the widespread occurrence of this virulence mechanism in bacteria isolated from the uterus of mares (Figure 1). Among the producers, 43.75% (7/16) were classified as weak producers, 31.25% (5/16) as moderate, and 25% (4/16) as strong, indicating variations in the potential for biofilm formation among the isolates, a relevant characteristic for the persistence of uterine infections.

After treatment with ozonated sunflower oil at a concentration of 203.43 mEq of peroxide/kg, a significant reduction (p < 0.05) was observed in the biofilm mass produced by gram-negative bacteria, indicating effective disruption of the pre-formed biofilm. Statistical analysis revealed a significant difference between the initial biofilm mass and the biofilm mass after treatment. In contrast, although a reduction in biofilm mass was also observed for gram-positive cocci after exposure to ozonated oil, this difference was not statistically significant when compared to the initial biofilm. In the control groups, treatment with extra virgin sunflower oil resulted in an increase in biofilm mass for gram-positive bacteria, while gram-negative bacteria did not show statistically significant changes compared to baseline values ​​(Table 2).

**Table 2:** Action of ozonated sunflower oil on biofilm of bacteria isolated from the uteruses of susceptible mares.

**Isolates**	**Initial biofilm**	**Biofilm after treatment**	**Biofilm control**
**Gram-positive cocci**	0.197^a^	0.128a	0.393ab
*Staphylococcus spp.*	(CI: 0.129 – 0.265)	(CI: 0.100 – 0.156)	(CI: 0.337 – 0.449)
*β-hemolytic Streptococcus*			
**Gram-negative bacilli**	0.161^a^	0.100^ab^	0.174^a^
*E. coli*	(CI: 0.135 - 0.188)	(CI: 0.081 - 0.120)	(CI: 0.127 - 0.222)
*Klebsiella* spp.			
NLF gram-negative bacilli			
*Proteus spp.*			

NLF: Non-lactose fermenting; CI: Confidence interval. ^a^No statistical difference found; ^ab^Statistical difference found (p < 0.05).

## Discussion

In mares with impaired uterine defense mechanisms, infectious endometritis is the main cause of infertility, being responsible for the inability to conceive in 25% to 60% of affected mares (Troedsson, 2011; Canisso, et al., 2020; Díaz-Bertrana et al., 2021). In the present study, 75.6% of the mares showed positive microbiological isolation. Oliveira et al. (2010), working with herds of mares from the *Agreste* and *Zona da Mata* regions in the state of Pernambuco, observed that 64% of the samples were positive in the microbiological assay, but with a smaller number of animals (n = 25) than in the present study. Cabrera et al. (2016) reported results closer to those observed in the present study, with positive cultures in 71.4% of the samples collected from 196 mares.

Of the 21 isolates submitted to biofilm production, 76.2% (16/21) exhibited positive production, among which 43.75% (7/16) were weak producers, 43.75% (7/16) moderate producers, and 12.5% (2/16) strong producers. Noel et al. (2016), when assessing the same capacity based on *Staphylococcus* spp. isolates from the milk of cows with mastitis, reported similar results, with 74.4% positive isolates, 65% of which were classified as strong producers and 35% as weak producers, varying according to the *Staphylococcus* species involved. Beehan et al. (2016), assessing the biofilm-formation capacity of 56 *E. coli* samples in isolates from mare uteruses, observed that 31% of the samples were considered strong producers, 8% moderate producers, and 61.5% weak producers.

The ability of a given microorganism to produce biofilms depends directly on the acquisition of genes that confer this ability (O’Toole et al., 2000; Nasr et al., 2012). According to Ferris et al. (2014), when bacteria are in a biofilm lifestyle, the tendency to acquire these genes increases as the exchange of genetic material is intensified, which allows for rapid horizontal transfer of genetic material. This makes the biofilm a perfect medium for the emergence of more resistant pathogens, through the acquisition of new mechanisms, such as the multidrug efflux pump. Furthermore, bacteria enveloped by biofilms are protected by structural factors, such as the extracellular matrix itself, which acts as a diffusion barrier, reducing the penetration of antibiotics into deeper layers, and physiological factors, such as metabolic reduction or inactivation, which decreases susceptibility to antibiotics that act on active cellular processes (Liu et al., 2024; Almatroudi, 2025; Abbas et al., 2025). Therefore, evaluating biofilm formation capacity is a very relevant aspect for understanding bacterial virulence and the persistence of infections, since microorganisms capable of forming biofilms tend to exhibit greater resistance to antimicrobial therapies and a greater capacity for survival in different biological environments.

The results of the present work showed a 2:1 ratio between the MBC and MIC for the large majority of bacteria assessed, with the exception of *Proteus* spp., for which the ratio was 4:1. Díaz et al. (2006), assessing the physicochemical and microbiological characteristics of sunflower oil, at 862 mEq peroxide/kg, and olive oil, observed MBC:MIC ratios of 5:1 for *Staphylococcus aureus* and 2.3:1 for *E. coli*. The same study reported an 11.7:1 MBC:MIC ratio when assessing oils with 2.506 mEq peroxide/kg. These results demonstrate that the higher the peroxide concentration in an oil after ozonation, the greater its antimicrobial capacity. This finding also justifies the difference between the ratios found by Díaz et al. (2006) and those in the present study, since the concentration of the oil used herein was 406.87 mEq peroxide/kg. No studies were found in the literature that justify the finding for *Proteus* spp., which was more sensitive to different concentrations of ozonated oil. However, the individual characteristics of each microorganism when faced with antimicrobial agents and conditions, which can vary among individuals of the same group, genus, and species, must be taken into account.

The antimicrobial effects of ozonized sunflower oil are mainly associated with the generation of oxidative species derived from the ozonation process. These compounds can induce lipid peroxidation of cell membranes, oxidation of structural proteins, and oxidative damage to microbial DNA, compromising cellular integrity and leading to bacterial death. Furthermore, the gradual release of these reactive species favors the degradation of the polymeric extracellular matrix of the biofilm, facilitating the structural disorganization of these microbial communities and increasing the exposure of bacterial cells to antimicrobial agents (Vieira et al., 2025; Araújo et al., 2024).

Ozonated sunflower oil at a concentration of 203.43 mEq/L was only effective in reducing biofilm mass in gram-negative bacilli, with statistically significant (p < 0.05) differences observed between the preformed biofilm and the biofilm after exposure to ozonated oil. These findings contrast with those reported by Ferris et al. (2014), who, when evaluating the efficacy of non-antibiotic agents, including ozonated saline, observed minimal biofilm reduction in *Escherichia coli* and *Klebsiella pneumoniae*, and no reduction in *Pseudomonas aeruginosa* isolated from horse uteruses. The authors attributed these results to the high volatility of O_3_, which dissipates rapidly at room temperature, probably before the bacteria are effectively exposed to the ozonated saline. In contrast, during the ozonation of vegetable oils, such as sunflower oil, O_3_ reacts almost exclusively with the carbon-carbon double bonds of unsaturated fatty acids. This reaction leads to the formation of 1,2,3-trioxolanes, which rapidly decompose into carbonyl compounds and aldehydes, that recombine to generate ozonides, aldehydes, hydroxyperoxides, and peroxides (Schwartz et al., 2011). When stabilized in oils, O_3_ is converted primarily to peroxides, which remain active for long periods, preserving their antimicrobial properties. This chemical stability likely accounts for the higher biofilm-disintegrating activity observed in the present study.

It was also observed that ozonated sunflower oil did not show the same level of efficacy against biofilms formed by gram-positive bacteria. Although a reduction in biofilm mass was observed after exposure, this decrease was not statistically significant (p > 0.05) when compared to the initial biofilm. Previous studies have shown that peptidoglycans, particularly N-acetylglucosamine, are resistant to O_3_ under acidic to neutral pH conditions (Perez et al., 1995). Therefore, the higher concentration of these molecules in the cell walls of gram-positive bacteria may explain the reduced effectiveness of ozonated oil in disrupting these biofilms. On the other hand, the greater susceptibility of gram-negative bacteria could be attributed to their thinner and less rigid cell walls and the presence of abundant lipoproteins and lipopolysaccharides (Thanomsub et al., 2002), which can facilitate O_3_ penetration, especially when O_3_ is supplied in the form of stabilized peroxides in vegetable oils. In the control group, which consisted of biofilms of gram-positive and gram-negative bacteria exposed to extra-virgin sunflower oil, an increase in biofilm mass for gram-positive bacteria was observed after treatment. This result may be associated with the presence of Tween 80, an emulsifier used to facilitate the dispersion of ozonated sunflower oil in Mueller-Hinton broth. Nielsen et al. (2016) reported that the addition of Tween 80 to *Staphylococcus aureus* cultures increased bacterial growth rates and resulted in a higher total biofilm mass *in vitro*. However, the same study found that Tween 80 had no effect on the growth of *Listeria monocytogenes* or *Pseudomonas fluorescens* and, in fact, reduced biofilm formation in these species.

## Conclusion

From the results of the current study, it can be concluded that the bacteria isolated from mares with endometritis were able to form biofilm *in vitro*. Ozonated sunflower oil showed antibacterial activity, inhibiting microbial growth at 101.71 mEq of peroxide/kg and promoting bacterial destruction at 203.43 mEq of peroxide/kg. At this concentration, the oil proved effective in degrading biofilms of gram-negative bacteria, but with low efficiency against gram-positive bacteria. The effectiveness of sunflower oil is related to its peroxide concentration, and further studies are needed to elucidate its mechanisms of action and effects on different microorganisms.

## Data Availability

Research data is only available upon request.
